# Risk of Spine Surgery in Patients with Rheumatoid Arthritis: A Secondary Cohort Analysis of a Nationwide, Population-Based Health Claim Database

**DOI:** 10.3390/medicina58060777

**Published:** 2022-06-08

**Authors:** Chien-Han Chen, Chia-Wen Hsu, Ming-Chi Lu

**Affiliations:** 1 Division of Obstetrics and Gynecology, Dalin Tzu Chi Hospital, Buddhist Tzu Chi Medical Foundation, Dalin, Chiayi 62247, Taiwan; dm500307@tzuchi.com.tw; 2 Department of Medical Research, Dalin Tzu Chi Hospital, Buddhist Tzu Chi Medical Foundation, Dalin, Chiayi 62247, Taiwan; chiawen0114@yahoo.com.tw; 3 Division of Allergy, Immunology and Rheumatology, Dalin Tzu Chi Hospital, Buddhist Tzu Chi Medical Foundation, Dalin, Chiayi 62247, Taiwan; 4 School of Medicine, Tzu Chi University, Hualien City 97004, Taiwan

**Keywords:** rheumatoid arthritis, surgery, spine, lumbar vertebrae

## Abstract

*Background and Objectives*: To study the risk of spine surgery, including cervical and lumbar spine surgeries in patients with rheumatoid arthritis (RA) compared with those without a diagnosis of RA. *Materials and Methods*: This is a secondary data analysis using population-based health claim data. We identified newly diagnosed adult patients with RA between January 2000 and December 2012, according to the International Classification of Diseases, Ninth revision, clinical modification code 714.0 from Taiwan’s National Health Insurance Research Database. Using data frequency-matched by 10-year age intervals, sex and index year with the RA cohort at a ratio of 5:1, we assembled a comparison cohort. All patients were followed until the study outcomes occurred (overall spine surgery, cervical spine surgery, or lumbar spine surgery) or the end of follow-up. Adjusted incidence rate ratios (aIRR) were calculated using Poisson regression analysis with age group, socioeconomic status, geographical region, and osteoporosis included as potential confounders. *Results*: We identified 1287 patients with RA and 6435 patients without RA. The incidence of overall spine surgery (aIRR = 2.13, 95% confidence interval (CI) = 1.49–3.04) and lumbar spine surgery (aIRR = 2.14, 95% CI = 1.46–3.15) were all significantly higher in the RA cohort. Moreover, females over 45 years of age were particularly at risk for lumbar spine surgery. In RA patients, older age and the combination with the diagnosis of osteoporosis had an elevated risk for overall and lumbar spine surgery. *Conclusion:* Patients with RA had an increased risk of receiving spine surgery. Physicians should be vigilant for possible spinal problems in women and older patients with RA.

## 1. Introduction

Rheumatoid arthritis (RA) is a common systemic inflammatory autoimmune disease characterized by painful peripheral joints. The chronic inflammation can lead to joint destruction that can severely impair physical function in patients with RA. The worldwide prevalence of RA is 0.5–1.0% with a female-to-male ratio of approximately 2.5:1 [1]. The most common age of RA occurrence is 40–70 years old, and the incidence of RA increases with age [2]. The prevalence of RA in Taiwan was around 0.26–0.93% and patients with RA show a higher mortality rate compared with controls [3,4].

In addition to involving peripheral joints, RA could also affect the spine. Patients with RA can have some unique cervical spine disorders, including C1-2 instability, basilar invagination, and subaxial subluxation [5]. However, whether these cervical spine abnormalities would cause an increased risk of receiving cervical spine surgery in patients with RA is not clear. It should be noted that patients with RA had increased prevalence of spondylolisthesis and vertebral fracture compared with controls [6]. The increased rates of spondylolistheses might be attributed to the facet erosion and osteoporosis, and female patients with RA and those with elevated serum levels of C-reactive protein were at risk for developing lumbar spondylolisthesis [7,8]. Studies using magnetic resonance imaging (MRI) showed that lumbar endplate and facet erosion are common in patients with RA [9]. However, whether these conditions were associated with an elevated risk of receiving lumbar spine surgeries in patients with RA is still unknown. Therefore, the aim of this secondary cohort study was to investigate the risk of receiving overall spine surgery, cervical spine surgery, and lumbar spine surgery in patients with RA compared with those without RA using data from a nationwide health claim database.

## 2. Materials and Methods

### 2.1. Identification of the Rheumatoid Arthritis and Comparison Cohort

This is a secondary data analysis using population-based health claim data. The study protocol was reviewed and approved by the institutional review board of the Dalin Tzu Chi Hospital, Buddhist Tzu Chi Medical Foundation, Taiwan (No. B10004021, 21 December 2018). As the NHIRD files contain only deidentified secondary data, the requirement for obtaining informed consent from individual patients was waived. The source of our data is the claim data from the Taiwan’s National Health Insurance Research Database (NHIRD).

The selection of patients in the RA cohort and the comparison cohort followed the protocol of our previous study, with some modifications [10]. Patients with newly diagnosed RA classified according to the International Classification of Diseases, ninth revision, clinical modification (ICD-9-CM) code 714.0 were identified from the 2000–2012 catastrophic illness datafile, which is a subset of the NHIRD. In Taiwan, to receive a waiver for medical co-payment, patients with RA can apply for a catastrophic illness certificate from the National Health Insurance Administration. The certification is issued to patients after their medical records, laboratory data, and imaging findings have been reviewed by at least two rheumatologists based on the American Rheumatism Association 1987 revised criteria [11]. In this study, the date of the application of the catastrophic illness certificate was defined as the index date for the RA.

A random sample of the outpatient datafile of the 2000 Longitudinal Health Insurance Database (LHID 2000) was used to assemble the comparison cohort. The LHID 2000 is a subfile of the NHIRD with health claim records between 1 January 2000 and 31 December 2012. Based on frequency matching for six 10-year age intervals, sex, and index year, five patients for each RA patient were selected.

Patients aged 20 to 80 years on the index date were included in the study. Patients who had been diagnosed with ankylosing spondylitis (ICD-9-CM code 720.0), or had received any spine surgery before the index data were excluded in both the RA and the comparison cohorts. Osteoporosis (ICD-9-CM code 733.0) was also included as a potential confounder in the regression analysis.

### 2.2. Identification of Cervical and Lumbar Spine Surgery

Patients in the RA and comparison cohorts were followed until the study outcomes had occurred or the end of follow-up period. Three study outcome variables were evaluated: overall spine surgery, cervical spine surgery, and lumbar spine surgery. Surgery codes were used to identify spine surgery from inpatient order datafiles. The codes included 64012B for costo-transversectomy; 64269B or 64270B for corrective osteotomy; 64144B or 64276B for curettage or excision of single or multiple vertebral body; 64160B for open reduction for fracture of spine; 64042C for close reduction for fracture of spine; 64279B for revisional diskectomy, cervical, thoracic, and lumbar; 64280B for revisional posterior spinal fusion with instrumentation, 83002C or 83003C for laminectomy; 83033C for laminoplasty; 83022C for cervical spine discectomy; 83024C for lumbar spine diskectomy; 83043B, 83044B, 83045B, 83046B, 83095B, 83096B, and 83097B for spine fusion;. Spine surgeries due to cancer (ICD-9-CM codes 140–239) and infection (ICD-9-CM codes 001–139), osteomyelitis (ICD-9-CM code 730), and intraspinal abscess (ICD-9-CM code 324) were excluded.

As the location of the surgery for most spine surgeries, except discectomy, is not specified by procedure codes, spine surgeries were classified into those related to the cervical or lumbar vertebrae based on the following ICD-9-CM diagnostic and procedure codes: ICD-9-CM procedure code 81.01, 81.02 and 81.03 for cervical spinal fusion; 81.04, and 81.05 for lumbar fusion, and 81.06, 81.07 and 81.08 for lumbar and lumbosacral fusion. Only spine surgeries that occurred 90 days after the index date were included in our analyses. A 90-day interval was used to lower the possibility of including spine surgeries that were not related to RA.

### 2.3. Statistical Analysis

The basic characteristics of the patients in the RA cohort and the comparison cohort were compared with the chi-square test for categorical variables and the t-test for continuous variables, as appropriate. The patients’ socioeconomic status was trichotomized into three groups based on their payroll-related insured amount, with cutoff points at TWD 19,000 and TWD 24,000 [12].

Incidence rate per 1000 person-years was calculated for the RA and the comparison cohorts, separately for each of the three outcome variables. Incidence rate ratios (IRR) and adjusted IRR with 95% confidence interval (CI) were calculated using generalized linear model with Poisson log-linear link function and person-years as the offset variable. Additional subgroup analyses were performed stratifying by sex or age groups (20–44, 45–59, and 60–80 years). All statistical analyses were conducted using IBM SPSS Statistics for Windows, version 24.0 (IBM Corp, Armonk, NY, USA). Two-tailed *p* values < 0.05 were considered statistically significant.

## 3. Results

**Figure 1:**
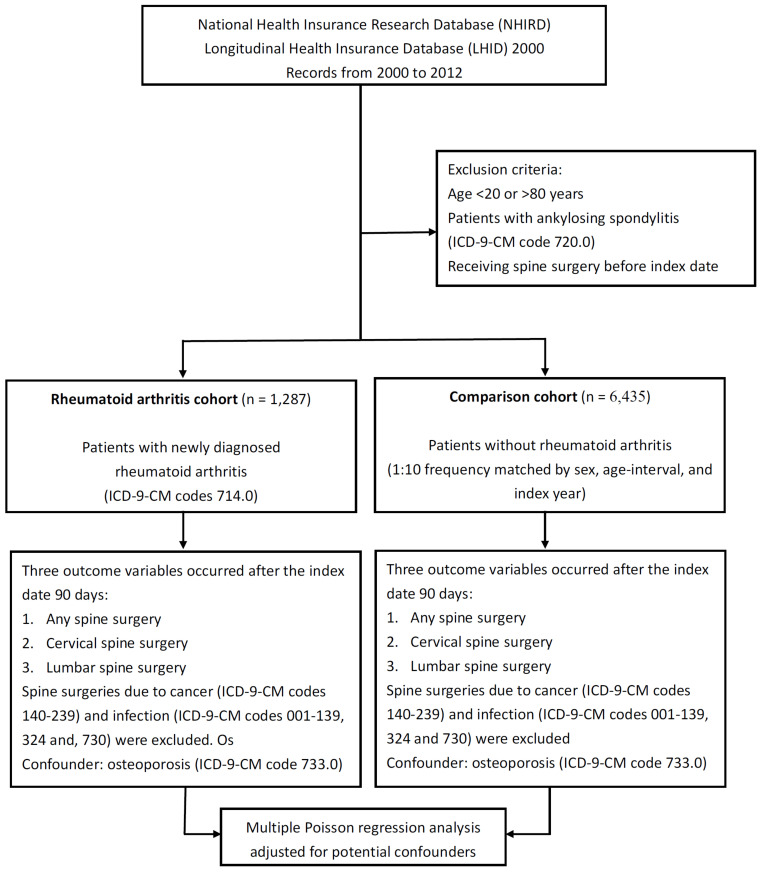
The flow chart for the study.

The study flow chart is shown in Figure 1.

Table 1 shows the basic characteristics of the patients in the RA cohort and the comparison cohort. A total of 1287 patients with newly diagnosed RA and 6435 patients without RA were selected from the database. The overall mean age was 53.4 years, with a standard deviation of 13.4 years. There were no significant differences between the two groups in sex (78.8% were females, *p* > 0.999) and age intervals (*p* > 0.999). In addition, there were no significant differences between the two cohorts in the distribution of geographic region (*p* = 0.455), but socioeconomic status was significantly higher in the RA cohort (*p* < 0.001). The prevalence of osteoporosis was also significantly higher in the RA cohort (*p* < 0.001).

The IRRs and adjusted IRR (aIRR) of overall spine surgery, cervical spine surgery, and lumbar spine surgery for the RA cohort and the comparison cohort, stratified by sex are shown in Table 2. For overall spine surgery, patients with RA (aIRR = 2.13; 95% confidence interval (CI) 1.49–3.04) and female patients with RA (aIRR = 2.43; 95% CI 1.65–3.58), but not male patients with RA showed a significantly higher incidence compared with the comparison cohort.

In addition, we stratified the location of spine surgeries into cervical and lumbar spine surgery. The location of spine surgery was determined using both ICD-9-CM diagnostic codes and procedure codes. After excluding those surgeries related to cancer, infection, osteomyelitis, and intraspinal abscess, we identified 50 spine surgeries in patients with RA, and 7 were located in the cervical spine, 42 in the lumbar spine and 1 in the thoracic spine. In the comparison cohort, 99 spine surgeries were identified, including 16 cervical spine surgeries, 82 lumbar spine surgeries, and 1 thoracic spine surgery. Two patients were receiving concurrent cervical and lumbar spine surgeries during the same admission course. We found that there was no significantly increased risk of cervical spine surgeries in patients with RA, either male or female patients. For lumbar spine surgery, patients with RA (aIRR = 2.14; 95% CI 1.46–3.15) and female patients with RA (aIRR = 2.44; 95% CI 1.61–3.69), but not male patients with RA, showed a significantly higher incidence compared with the comparison cohort.

IRs and IRRs of overall spine surgery, cervical spine surgery, and lumbar spine surgery for the RA cohort and the comparison cohort, stratified by three age groups (20–44 years, 45–59 years, and 60–80 years) are shown in Table 3. In overall spine surgery, while the IRRs were significant for all three age groups, the magnitude was the largest in the youngest age group (aIRR = 5.73; 95% CI 1.55–21.21) in the RA cohort compared with the comparison cohort. For cervical spine surgery, the risk of was elevated in the youngest age group with marginal significance (aIRR = 8.98; 95% CI 0.84–96.26, *p* = 0.070). For lumbar spine surgery, the risk ratio was significantly elevated in the age group 45–59 years (aIRR = 2.32; 95% CI 1.30–4.13) and the 60–80 years (aIRR = 1.90, 95% CI 1.10–3.29).

In Table 4, we analyzed the risks of overall spine surgery and lumbar spine surgery in patients with RA. In overall spine surgery, the IRRs were significantly elevated in age group 60–80 years (aIRR = 3.06; 95% CI 1.22–7.69) compared with age group 20–44 years and in those with the diagnosis of osteoporosis (aIRR = 3.06; 95% CI 1.22–7.69) compared with those without osteoporosis for patients with RA. In lumbar spine surgery, the IRRs were also significant elevated in age group 60–80 years (aIRR = 5.36; 95% CI 1.56–18.40) compared with age group 20–44 years and in those with the diagnosis of osteoporosis (aIRR = 2.80; 95% CI 1.46–5.36) compared with those without osteoporosis for patients with RA.

## 4. Discussion

Our cohort study showed that patients with RA exhibited a significantly increased risk of receiving spine surgery, especially over the lumbar spine but not the cervical spine. It should be noted that only seven cases receiving cervical spine surgery over 1287 patients with RA and 16 over 6435 controls were identified. The number of cases might be too small to reach statistical significance. According to a meta-analysis using 12 studies, long RA duration was a risk factor for cervical spine involvement [13]. RA cervical spine involvement in patients with RA has been reported to occur generally after 10 years of disease duration [14]. However, the mean follow-up period was only 6.04 years (standard deviation 3.63 years) for our patients with RA. Therefore, a larger sample and a longer follow-up duration are needed to further clarify the risk of cervical spine surgery in patients with RA.

In this study, we found that the risk of receiving lumbar spine surgery was significantly elevated in patients with RA, especially in female patients older than 45 years. Lumbar spine problems may have been overlooked in patients with RA [15]. In fact, abnormal radiologic findings in the lumbar spine were detected in 57% of patients with RA [16]. Suzuki et al. showed that patients with RA were more likely to have spondylolisthesis, vertebral fracture, and scoliosis compared with controls [6]. Our study also demonstrated that patients with RA had a higher risk of receiving lumbar spine surgery, even with adjustment for osteoporosis. Patients with RA receiving spine surgery had a three-fold increase in complications, such as radiographic evidence of nonunion, implant failure, symptomatic adjacent segment disease, and infection [17]. Female and older patients are already prone to having degenerative lumbar spondylolisthesis [18]. The participation of inflammation caused by RA might further accelerate the process [9].

There are several limitations in this study. First, due to the constraints imposed by the NHIRD database, the disease activity of RA, imaging, and serology reports were not available for analysis. Second, the identification of spine surgery was based on the diagnosis and procedure codes from the ICD-9-CM, which limits further investigation of the detailed surgery types. Third, as there is universal health coverage in Taiwan with low financial barrier for accessing medical care, including surgeries, our findings may not be generalizable to other populations based on different medical care systems. Finally, in addition to age, there are multiple other risk factors for developing spondylolisthesis including genetic, occupation, daily activity, sport, obesity and sedentary work [19,20]. This information was not available from the NHIRD database.

## 5. Conclusions

Patients with RA had an increased risk of receiving spine surgery, especially in the lumbar spine. Female and older patients with RA were at higher risk. Physicians should be vigilant for possible spinal problems in women and older patients with RA.

## Figures and Tables

**Table 1:** Basic characteristics of the patients in the rheumatoid arthritis cohort and the comparison cohort (*n* = 7722).

Variable	*n* (%)				*p*
	RA Cohort 1287 (16.7)		Comparison Cohort 6435 (83.3)		
Sex					>0.999
male	273	(21.2)	1365	(21.2)	
female	1014	(78.8)	5070	(78.8)	
Age group (years)					>0.999
20.0–29.9	58	(4.5)	290	(4.5)	
30.0–39.9	141	(11.0)	705	(11.0)	
40.0–49.9	297	(23.1)	1485	(23.1)	
50.0–59.9	376	(29.2)	1880	(29.2)	
60.0–69.9	235	(18.3)	1175	(18.3)	
70.0–80.0	180	(14.0)	900	(14.0)	
Mean age (SD), years	53.4	(13.4)	53.4	(13.4)	0.959
Mean follow up duration (SD), years	6.04	(3.63)	5.71	(3.86)	0.004
Osteoporosis	132	(10.3)	218	(3.4)	<0.001
Socioeconomic status (*n* = 7701)					<0.001
low	595	(46.6)	3714	(57.8)	
middle	456	(35.7)	1800	(28.0)	
high	225	(17.7)	911	(14.2)	
Geographic region (*n* = 7462)					0.455
northern	723	(58.4)	3740	(60.1)	
central	226	(18.2)	1030	(16.6)	
southern	262	(21.1)	1328	(21.3)	
eastern	28	(2.3)	125	(2.0)	

Socioeconomic status was estimated by insurance premiums based on salary. Low: ≤19,000 New Taiwan dollars (TWD); middle: 19,001–24,000 TWD; and high: >24,000 TWD. IQR: interquartile range; RA: rheumatoid arthritis; SD: standard deviation. *p* values were obtained by the chi-square test for categorical variables and the *t*-test for continuous variables, as appropriate.

**Table 2:** The incidence rate and incidence risk ratio of spine surgery in the rheumatoid arthritis cohort and the comparison cohort (*n* = 7722).

Outcome Variable	RA Cohort (*n* = 1287)			Comparison Cohort (*n* = 6435)			IRR (95% CI)	Adjusted IRR * (95% CI)
	No. of Patient	Person-Years	IR	No. of Patient	Person-Years	IR	*p*	*p*
**Spine surgery**								
total	50	7572	6.60	97	35,821	2.71	2.44 (1.73–3.43) <0.001	2.13 (1.49–3.04) <0.001
male	5	1531	3.27	23	7541	3.05	1.07 (0.41–2.82) 0.890	1.03 (0.38–2.81) 0.950
female	45	6041	7.45	74	28,280	2.62	2.85 (1.96–4.12) <0.001	2.43 (1.65–3.58) <0.001
**Cervical**								
total	7	7572	0.92	16	35,821	0.45	2.07 (0.85–5.03) 0.108	1.79 (0.68–4.71) 0.238
male	1	1531	0.65	7	7541	0.93	0.70 (0.09–5.72) 0.742	0.89 (0.11–7.44) 0.915
female	6	6041	0.99	9	28,280	0.32	3.12 (1.11–8.77) 0.031	2.27 (0.74–6.98) 0.153
**Lumbar**								
total	42	7572	5.55	82	35,821	2.29	2.42 (1.67–3.52) <0.001	2.14 (1.46–3.15) <0.001
male	4	1531	2.61	18	7541	2.39	1.10 (0.37–3.24) 0.870	0.99 (0.32–3.05) 0.989
female	38	6041	6.29	64	28,280	2.26	2.78 (1.86–4.15) <0.001	2.44 (1.61–3.69) <0.001

CI: confidence interval; IR: incidence rate per 1000 person-years; IRR: incidence rate ratio; RA: rheumatoid arthritis. * Adjusted for age group, socioeconomic status, geographic region, and osteoporosis.

**Table 3:** The incidence rate and incidence risk ratio of spine surgery in the RA cohort and the comparison cohort (*n* = 7722).

Outcome Variable	RA Cohort (*n* = 1287)			Comparison Cohort (*n* = 6435)			IRR (95% CI)	Adjusted IRR * (95% CI)
	No. of Patient	Person-Years	IR	No. of Patient	Person-Years	IR	*p*	*p*
**Spine surgery**								
Age group, years								
20–44.9	7	2050	3.41	4	9827	0.41	8.39 (2.46–28.66) 0.001	5.73 (1.55–21.21) 0.009
45–59.9	20	3362	5.95	43	15,938	2.70	2.20 (1.30–3.75) 0.003	2.22 (1.29–3.84) 0.004
60–80.0	23	2160	10.65	50	10,056	4.97	2.14 (1.31–3.51) 0.003	1.78 (1.06–2.98) 0.029
**Cervical**								
Age group, years								
20–44.9	4	2050	1.95	1	9827	0.10	19.17 (2.14–171.55) 0.008	8.98 (0.84–96.26) 0.070
45–59.9	2	3362	0.59	6	15,938	0.38	1.58 (0.32–7.83) 0.575	2.07 (0.40–10.70) 0.387
60–80.0	1	2160	0.46	9	10,056	0.89	0.52 (0.07–4.08) 0.532	0.50 (0.06–4.07) 0.516
**Lumbar**								
Age group, years								
20–44.9	3	2050	1.46	3	9827	0.31	4.79 (0.97–23.75) 0.055	4.58 (0.91–23.13) 0.066
45–59.9	18	3362	5.35	37	15,938	2.32	2.31 (1.31–4.05) 0.004	2.32 (1.30–4.13) 0.004
60–80.0	21	2160	9.72	42	10,056	4.18	2.33 (1.38–3.93) 0.002	1.90 (1.10–3.29) 0.022

CI: confidence interval; IR: incidence rate per 1000 person-years; IRR: incidence rate ratio. * Adjusted for sex, socioeconomic status, geographic region, and osteoporosis.

**Table 4:** The risk factors for spine surgery and lumbar spine surgery in the RA cohort (*n* = 1287).

	Overall Spine Surgery		Lumbar Spine Surgery	
Variable	aIRR * (95% CI)	*p*	aIRR * (95% CI)	*p*
Sex (female/male)				
male	Ref		Ref	
female	2.16 (0.85–5.51)	0.107	2.27 (0.80–6.45)	0.123
Age group (years)				
20–44.9	Ref		Ref	
45–59.9	1.85 (0.74–4.63)	0.192	3.23 (0.94–11.05)	0.062
60–80	3.06 (1.22–7.69)	0.018	5.36 (1.56–18.40)	0.008
Osteoporosis				
No	Ref			
Yes	2.34 (1.25–4.38)	0.008	2.80 (1.46–5.36)	0.002
Socioeconomic status				
low	Ref		Ref	
Middle and high	1.07 (0.60–1.91)	0.816	1.07 (0.57–2.00)	0.836
Geographic region				
northern	Ref		Ref	
central, southern, and eastern	1.13 (0.64–2.00)	0.678	0.97 (0.52–1.80)	0.916

CI: confidence interval; aIRR: adjusted incidence rate ratio; * Adjusted for sex, age group, osteoporosis, socioeconomic status, and geographic region.

## Data Availability

Availability of data and material: The data that support the findings of this study are available on request from the corresponding author. The data are not publicly available due to the Taiwan Personal Information Protection Act.
